# Effects of Heated Drinking Water on the Growth Performance and Rumen Functionality of Fattening Charolaise Beef Cattle in Winter

**DOI:** 10.3390/ani11082218

**Published:** 2021-07-27

**Authors:** Silvia Grossi, Luciana Rossi, Matteo Dell’Anno, Stefano Biffani, Carlo Angelo Sgoifo Rossi

**Affiliations:** 1 Department of Health, Animal Science and Food Safety “Carlo Cantoni” (VESPA), Università Degli Studi di Milano, 26900 Lodi, Italy; luciana.rossi@unimi.it (L.R.); matteo.dellanno@unimi.it (M.D.); carlo.sgoifo@unimi.it (C.A.S.R.); 2 Consiglio Nazionale delle Ricerche (CNR), Istituto di Biologia e Biotecnologia Agraria (IBBA), Via Edoardo Bassini, 15, 20133 Milano, Italy; biffani@ibba.cnr.it

**Keywords:** rumen kinetics, rumen health, production efficiency, drinking water

## Abstract

**Simple Summary:**

Efficiency has been a major metric for measuring the production performances and profitability of zootechny, especially in beef cattle farming, even in terms of environmental sustainability. More efficient beef cattle farming should include a decrease in total feed consumption, equal or greater production performance and lower methane emissions and manure excretion. In fact, the rumen is the keystone of cattle efficiency. Strategies to maintain the rumen’s stability while enhancing its functionality can be drivers of better overall efficiency. Besides the feeds used and nutritional management, drinking water—specifically, its temperature—can alter and modulate the ruminal environment, due to the high sensitivity of the ruminal microbiota. Drinking heated water kept at a constant temperature can lead to enhanced production efficiency, due to better rumen stability and increased viability of ruminal bacteria.

**Abstract:**

The effects of heated drinking water on growth performance and rumen functionality in fattening beef cattle during winter were evaluated. Newly received Charolaise bulls (*n* = 224) were allocated to two experimental groups: (i) water at room temperature (RTW) (weight 408 ± 34 kg); (ii) constantly heated water (25 °C) (HW) (weight 405 ± 38 kg). Growth performances, feed intake, feed conversion rate, water intake and carcass characteristics were evaluated. Internal reticuloruminal wireless boluses were used to collect rumen pH and temperature values every 10 min. Bodyweight was not affected by the water temperature, but the overall average daily gain (ADG) was significantly higher in the HW group (1.486 vs. 1.438 kg/head/day in the RTW; *p* = 0.047). Dry matter intake was significantly higher in the HW group (*p* = 0.001), even though the final feed conversion rate (FCR) was not influenced. There was also a tendency for better cold carcass weight (CCW) and carcass yield (CY) in the HW group. Drinking heated water reduced the time (min/day) during which the ruminal pH was below pH 5.8 or 5.5, and the time during which the temperature was lower than 37 or 39 °C (*p* < 0.001). The use of heated drinking water is a plausible a strategy for enhancing ruminal stability and the overall production efficiency in fattening beef cattle, which will lead to both better growth performance and higher ruminal stability.

## 1. Introduction

Feed efficiency is a major metric for measuring the production performances and profitability of poultry, swine and cattle. In recent decades, the concept of sustainable agriculture emerged, which has the overall aims of safeguarding natural resources, promoting a clean environment and improving both producer profitability and animal welfare. Even in terms of environmental sustainability, feed efficiency has become a valuable parameter in livestock production [1,2,3,4,5]. Superior feed efficiency in beef cattle farming would mean decreasing feed consumption while maintaining greater or equal production performance and producing less methane and manure [6]. Therefore, improving feed efficiency has the potential to simultaneously increase profitability while reducing the environmental impacts of cattle production [7,8,9].

The rumen and its health are the key to improving production efficiency in beef cattle farming [10]. The rumen is a complex microbial environment, hosting numerous strains of bacteria and microorganisms which coexist and perform at best only in specific and stable environmental conditions [11]. The main factors influencing the growth and activity of ruminal microbiota are pH (average values of 5.8–6), buffering capacity, osmotic pressure and redox potential. In addition, rumen temperature can affect ruminal functionality [12]. Indeed, rumen temperature has to be maintained in the range of 39 to 39.5 °C [13]. A decrease in rumen temperature can alter microbial activity, reducing both the number of microbes and bacterial diversity, richness and functionality, leading to both poorer overall dry matter digestibility and poorer production of short-chain fatty acids [14]. Specifically, deviations in rumen temperature may impair fiber digestion [15].

Several studies investigated the effect of water temperature on rumen functionality in dairy cows [16,17,18]. In dairy cows, cold water intake can drastically decrease the ruminal temperature for extended periods. In one study, there was a deep and sudden decline that required more than three hours for recovery. It had a negative impact on ruminal stability [16,17,18]. Moreover, a higher average water intake was recorded in dairy cows supplied with heated water during winter, compared to cows supplied with room-temperature water [17,19]. Due to the strong positive relationship between water and solid feed intake, greater water intake can lead to greater feed consumption [19]. Indeed, the administration of heated water can be a strategy for enhancing rumen stability, feed intake and feed efficiency.

Few scientific studies have investigated the effects of drinking water temperature on beef cattle efficiency. However, some positive effects on growth performance were reported when heated water was administered in the winter period [20,21].

No studies have investigated heated versus cold drinking water in terms of their effects on both growth performance (e.g., average daily gain) and rumen parameters (e.g., pH and temperature) in beef cattle. A step forward in understanding the effects of water temperature on beef cattle feed efficiency could be promoted by using precision livestock farming technologies, such as wireless telemetry [22].

Indeed, wireless telemetry technologies have been applied to develop boluses for monitoring the rumen’s pH and are now often used to control/measure the physiological parameters that can indicate livestock diseases, including the detection of subacute ruminal acidosis, and other variations in the ruminal environment. Therefore, this technology improves the timeliness of disease detection and contributes to safeguarding animal health and productivity [23]. Continuous rumen pH and temperature measurements may be useful tools for evaluating the effects of water temperature on ruminal parameters in cattle [16,24].

The aim of this study was to investigate the potential effects of water temperature on growth performance, efficiency and ruminal functionality, using continuous reticuloruminal indwelling boluses, in Charolais fattening beef cattle.

## 2. Materials and Methods

### 2.1. Animals, Groups and Animal Care

The study was performed in a commercial intensive beef fattening farm which is representative of the Italian intensive beef cattle farming system.

A total of 224 newly received Charolaise bulls, imported from France, were enrolled. At three days after arrival (d_0_), all the animals were individually weighed, and conformation was assessed on a 5-point scale [25]. The animals were grouped by conformation and body weight, and randomly assigned to two balanced experimental groups, differing by the drinking water temperature: (i) drinking water at room temperature (RTW) (initial weight 408 ± 34 kg); (ii) heated water at a constant temperature of 25 °C (HW) (initial weight 405 ± 38 kg). The trial lasted for the entire fattening period (180 days).

The bulls were housed in a close barn with 32 pens containing 7 animals each (3.5 m^2^ each) and a fully slatted floor.

The barn facilities allowed us to administer drinking water at two different temperatures. In fact, the pens were arranged in two parallel lanes, allowing us to differentiate the water supply systems and lines. The two groups received their drinking water from two different and separate supply systems, to allow the heating for the HW group.

All the animals had free access to the water that was administered ad libitum through drinking bowls (1 for each pen),

The water was heated to 25 °C using a continuous heating machine powered by electricity (CMP Technologies—Viadana, Bresca, Italy). The temperature of the water for the RTW group was monitored monthly, using a thermometer. The average temperature values are reported in Table 1.

All the animals were inspected twice a day by the veterinary staff of the farm, and in cases of disease were treated following the standard farm sanitation protocols.

### 2.2. Feeding Protocol

All the groups received the same feeding plan (Table 2). The feed was administered in the form of total mixed ration (TMR), administered ad libitum and delivered once a day in the morning by a feed mixer wagon, which had an electronic scale to weigh the inclusion of each ingredient and the TMR as it was eaten. The TMR was studied to meet the growth needs of the animals, as required by the Nutrient Requirement Council [26]. The fattening period was divided into 4 subperiods to better fit the different needs of every growing phase: arrival (0–30 days), base (30–60 days), fattening (60–130 days), finishing (130–180 days).

The two experimental groups differed by the drinking water temperature—heated constantly to 25 °C in the HW group and maintained at room temperature in the RTW group.

### 2.3. Experimental Parameters

#### 2.3.1. Growth, Feed and Water Intake, Feed Conversion Rate and Slaughtering Performances

Individual body weight was recorded before morning feeding three times: on the enrolment day (d_0_), on day 90 (d_90_) and on the day before slaughter (d_180_). The individual average daily gain (ADG) from d_0_ to d_180_ was then calculated as

$$ADG=\frac{Weight_{180}-Weight_{0}}{days\;of\;feed}$$

Pen feed intake was registered daily. The daily discharge and the residue were weighed and recorded for each pen, and then divided by the number of bulls in each, to obtain the individual feed intake. The average daily feed intake and the average daily gain were used to calculate the feed conversion rate (FCR). FCR was estimated as:

$$FCR=\frac{Group\;daily\;feed\;intake,\;kg}{Average\;daily\;gain,kg/head/day}$$

The water intake of the experimental group was also measured on a daily basis. Two water flowmeters were placed on the two water supply lines. Every day, the values were read, and water intakes of the experimental groups were calculated. The individual water intake was then calculated by division using the number of bulls in each experimental group.

At the slaughterhouse, cold carcass weights (CCW), carcass yields (CY) and SEUROP carcass rating scores (SCRS) were recorded for all the animals.

#### 2.3.2. Rumen Environment Health and Functionality

The reticuloruminal pH and temperature were monitored through an indwelling and wireless data transmitting system using on four animals per group (SmaXtec animal care GmbH, Graz, Austria). The system consists of wireless, indwelling rumen pH and temperature monitoring boluses (SmaXtec Premium Bolus SX-1042A); a base station that allows the boluses to be read and the data to be transferred to the software; and a program that collect and analyses all data. According to the manufacturers’ instructions, firstly all devices were calibrated, and successively the boluses were administered through a balling gun by mouth to each animal [27]. The boluses captured data about rumen pH and temperature every 10 min. The technical data from the SmaXtec Premium Bolus SX-1042A are reported in the Supplementary Table (Table S1).

Daily mean, minimum and maximum pHs were recorded. Moreover, following Penner et al. (2007), the total amounts of time spent below ruminal pHs 5.8 and 5.5 were also evaluated [28]. The same measurements and observations were also made for rumen temperature, using and 37 and 38 °C as thresholds for cold stress in the rumen.

Those data were recorded for a period of five months (from November to March).

### 2.4. Statistical Analysis

For all models, hypothesis testing and least-square means for fixed effects were performed and calculated using the ANOVA and the lsmeans functions from the stats (R Core Team 2018) and emmeans R packages, respectively. Mixed models were fitted using the LmerTest package [29]. Plots were created using package ggplot2 [30].

For all the parameters, a *p*-value ≤ 0.05 was considered statistically significant. A value ≤ 0.1 was considered a tendency.

#### 2.4.1. Growth Performance

Firstly, a Shapiro–wilk test was performed to check the normality of the data, and the homogeneity of variance was evaluated by fitting a Levene’s test. After that, the effect of water temperature (HW vs. RTW) on weight at times d_0_, d_90_ and d_180_ was analyzed using a Student’s *t*-test.

The effect of the experimental group (HW vs. RTW) on ADG during the two periods separately, namely, from d_0_ to d_90_ and from d_90_ to d_180_, and across the entire fattening period, from d_0_ to d_180_, was evaluated using a mixed model. The fitted model included a fixed-time linear regression nested within each experimental group and a random bull effect. The latter was used to model the covariance between repeated measures of the same individual. Least-square means for each model were also calculated.

#### 2.4.2. Feed Intake and Feed Conversion Rate

A mixed model was also used to investigate the effect of the experimental group (HW vs. RTW) on FCR, estimated as the ratio between group feed intake and the individual weight daily gain. The model included a fixed time linear regression nested within each experimental group and a random bull effect to model repeated observations per individual. Data about feed intake were analyzed using SAS statistical software (SAS 9.4, SAS Cary NC, USA). These data were analyzed using a mixed model (PROC MIXED) which considered the fixed effect of the treatment and the random effect of the day of recording.

#### 2.4.3. Slaughtering Performance

A linear model was used to analyze the effects of the experimental group on both CCW and CY. The model included the experimental group’s effect weight and the linear regression of initial weight. Moreover, the frequencies of SEUROP carcass rating scores (SCRS) by experimental group were calculated. Then both a Chi-squared test and Kruskal–Wallis tests were used to investigate possible differences in SCRS frequency due to experimental groups.

#### 2.4.4. Water Consumption

Water consumption per week was compared by experimental group, fitting a mixed model which included the fixed effect of the interaction between groups and calendar month, and the random effect of the week nested within each experimental group.

#### 2.4.5. Rumen pH and Temperature

Rumen pH and temperature were compared by experimental group, fitting a mixed model which included the fixed effect of the interaction between groups and calendar month, and the random effect of the week nested within each experimental group.

## 3. Results

### 3.1. Growth, Feed Intake, Feed Conversion Rate and Slaughtering Performance

**Figure 1:**
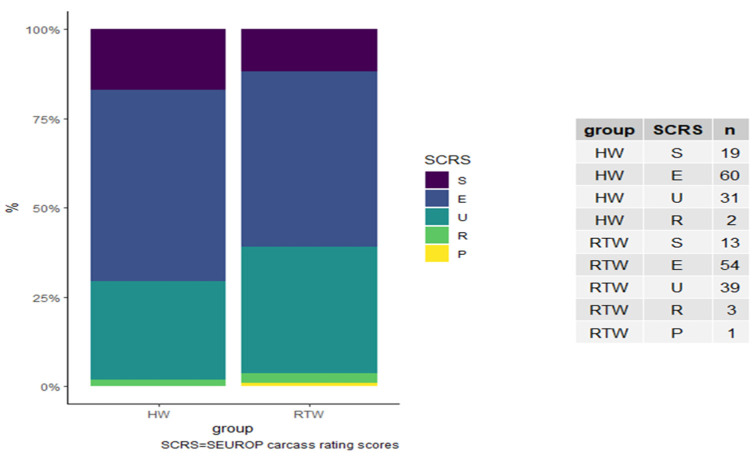
Frequencies of SEUROP carcass rating scores (SCRS) in the two experimental groups.

Results and descriptive statistics for growth performance, feed intake, feed conversion rate and carcass characteristics are reported in Table 3 and Figure 1.

The initial live weight was not significantly different between the two groups (405 ± 38 kg and 408 ± 38 kg for HW and RW, respectively; *p* = 0.59), suggesting correct allocation on arrival. Additionally, the administration of heated water did not affect the average weights significantly at d_90_ (546 ± 40 kg and 544 ± 34 for HW and RW, respectively; *p* = 0.74) or at the end of the fattening period, d_180_ (719 ± 59 kg and 711 ± 57 kg for HW and RW, respectively; *p* = 0.28) (Table 2).

Conversely, the total average daily gain (ADG_0–180_) was significantly increased in the HW group (1.486 kg/head/day) compared to the RTW group (1.438 kg/head/day) *(p =* 0.047). When considering the intermediate values (ADG_0–90_ and ADG_90–180_), the results were not influenced by the treatment *(p =* 0.160 and 0.158, respectively).

The overall feed intake was significantly higher in the HW group than in the RTW group *(p <* 0.001), which is a possible explanation of the better total ADG. In reverse, the FCR was not influenced statistically by the treatment *(p =* 0.260), mainly due to the higher feed intake.

Considering slaughtering performances, there was a tendency for higher cold carcass weight in the HW group *(p =* 0.084). This result can be explained by both the higher final weight of HW animals and the significantly higher carcass yield *(p =* 0.059) of HW animals.

Results for SEUROP carcass rating scores (SCRS) are reported in Figure 1. A higher frequency of high scores (S and E) was allocated to the HW group compared to the RTW group, even though the difference was not significant.

### 3.2. Water Intake

**Figure 2:**
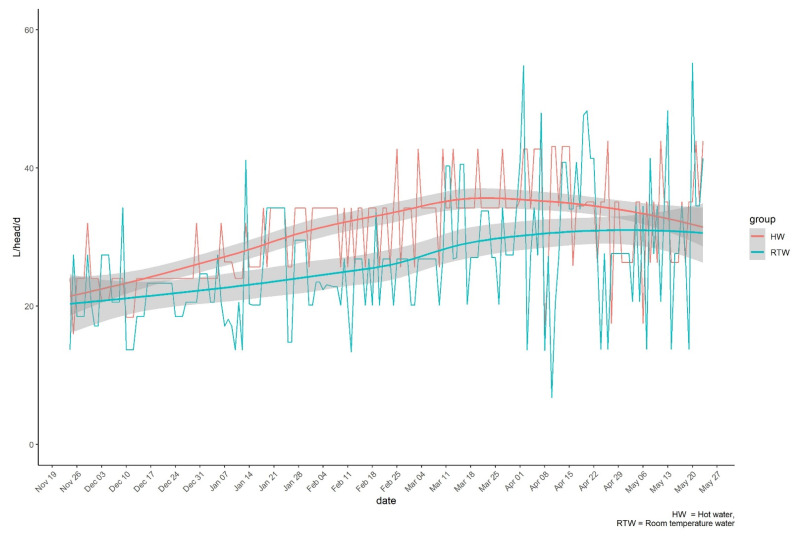
Trends in individual water consumption (L/head/d) during the trial in HW and RTW groups.

**Figure 3:**
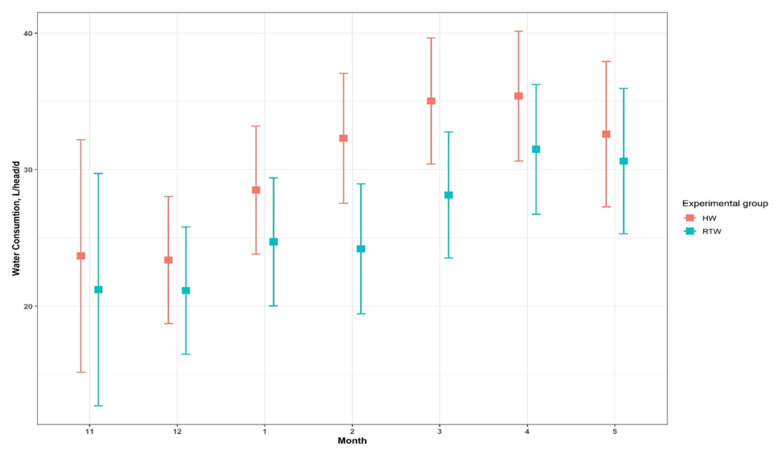
Water consumption (L/head/day) by month and experimental group.

The trends in daily water consumption by the HW and RTW groups over the trial period are presented in Figure 2 and Figure 3. Daily water consumption per head increased over the course of the trial in both groups. The intake in the two groups started to diverge during mid-January with higher consumption in the HW group than in the RTW group. On average, the daily water intake was 16% higher in the HW group than the RTW group (30.13 L/head/d vs. 25.93 L/head/d). Both group and month effects were statistically significant *(p =* 0.05). There was a tendency toward statistical significance *(p =* 0.1) for the interaction between month and group.

### 3.3. Rumen Environment Health and Functionality: pH

**Figure 4:**
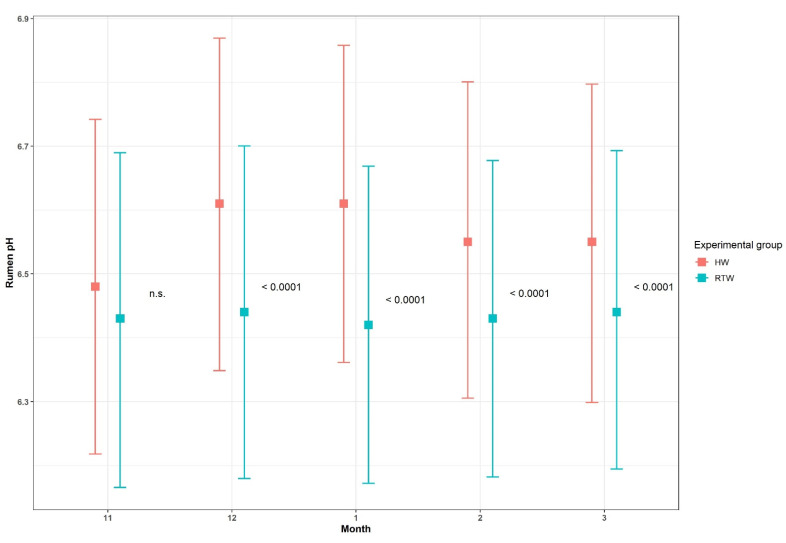
Rumen pH: the interaction between month and experimental group.

Results for ruminal pH are reported in Table 4. The interaction between month and experimental group was significant *(p <* 0.001): there were higher pHs during every period in HW-group cows than RTW-group cows. The least-square means for the month–group interaction, the 95% confidence intervals and the relative *p*-values are in Figure 4.

The experimental group had a significant effect on the amounts of time (min/day) that ruminal pH spent below 5.8 and 5.6 (Table 4). The ruminal pHs of the HW bulls lay under the thresholds of 5.8 and 5.5 for 34% *(p <* 0.001) and 29.9% *(p <* 0.001) less time compared to the RTW animals, respectively. The numbers of days when rumen pH remained below the relevant threshold (pH = 5.5 or 5.8) for at least 10 consecutive minutes were 14 and 41 out of 111 days for HW and RTW groups, respectively.

### 3.4. Rumen Health and Functionality: Temperature

**Figure 5:**
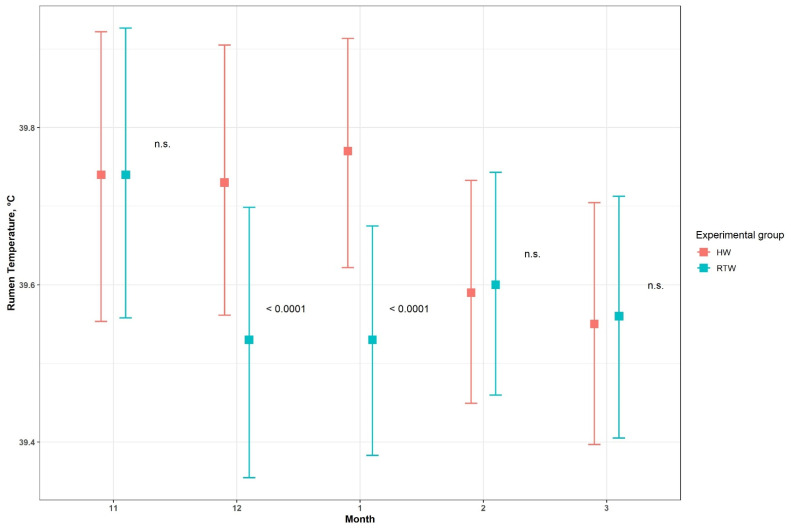
Rumen temperature: the interaction between month and experimental group.

Results for ruminal temperature (T°C) are shown in Table 5. The interaction between experimental group and month was significant *(p <* 0.001). However, the average RT for the HW group was statistically higher than for the RTW group only in December and January, the coldest month of trial, suggesting the importance of using heated water in the coldest periods (Figure 5).

Finally, the duration (min/day) that ruminal T°C was below 38 or 37 °C was positively and significantly affected by the water temperature, as shown in Table 5. The ruminal T°C of the HW bulls lay under the thresholds of 38 and 37 °C for 46.8% *(p <* 0.001) and 68.1% *(p <* 0.001) less time compared to the RTW animals. The total numbers of days when rumen T°C experienced a temperature lower than the designated threshold (38 or 37 °C) for at least 10 consecutive minutes were 109 and 111 out of 111 days for HW and RTW groups, respectively.

## 4. Discussion

The aim of the present study was to evaluate the effects of heated drinking water at a constant temperature of 25 °C on growth performance and ruminal parameters. The overall goal was to find a strategy to enhance production efficiency in beef cattle farming. Indeed, a healthy rumen is related to better and more efficient use of the feed for growth and production purposes, and reductions in noxious emissions, such as greenhouse gases and nitrogen. Hence, enhancing both rumen stability and rumen efficiency is crucial to improving the productivity, health, welfare and economic and environmental sustainability of beef cattle.

The two main parameters which can influence rumen health, through actions on its microbiota, are pH and temperature, both in their absolute values and daily variations. Those parameters can be modulated by many factors connected to nutrition, managing, behavior and individuality [24,31]. The rumen can also be affected by the characteristics of the drinking water, specifically by its temperature [16,17]. In fact, the results of the present study highlighted some positive effects of administering heated water at a constant temperature of 25 °C to beef cattle, compared to the intake of room temperature water during the winter season, in terms of both growth performance and ruminal parameters.

Regarding growth performance, the results of the present study highlighted a positive effect on ADG (+3.5%) in the HW group. There was a statistically significant difference, even though only the final weight was higher (+8 kg). Those findings partially agreed with previous studies, even though the magnitude of the effect was lower in the present study [20,21]. Chen Zhaohui et al. (2015) reported an increase of 30% in average daily gain (ADG) after the administration of heated drinking water (>17 °C) to beef cattle in the winter; this was in opposition to water <5 °C [21]. Additionally, Diao et al. (2012), in similar experimental conditions, reported positive results of heated drinking water (20 °C) in ADG (+13.5%) in fattening beef cattle, in comparison to animals receiving water at 4 °C [20]. Those differences in efficacy can be ascribed to the harsh winter weather conditions recorded in those studies, unlike those recorded in Italy during the present study. Conversely, Osborne et al. (2002), did not find any improvement in production performance in dairy cows when heated water was administered during an entire year, even though the feed and water intake were improved [19]. This finding disagrees with the evidence that increases in water and feed intake normally lead to improvements in production performance because of more rumen fermentation and synthesis.

In the present study, the average dry matter intake was 690 g/head/day higher, statistically significantly, in the HW group (+13.3%) compared to the RTW group, which can be linked to the higher water consumption by the HW group. Sexson et al. (2012) showed that as water intake increased, the DMI increased in yearling steers reared in feedlot [32]. Steiger et al. (2001) reported that a reduction in water intake decreased milk yield by up to 26% [33], whereas Daros et al. (2019), demonstrated that free access to water increased milk production by up to 1.7 L/head/d [34].

Multiple factors affect water intake, such as the physiological condition of the animal, dry matter intake, water availability, the quality of the water and the ambient temperature. Water temperature is recognized as another factor that can influence animal preferences, and consequently water intake [17,35]. This relationship was confirmed in the present study. Animal in the HW group drunk 16% more water compared to animals in the RTW group, especially in the colder months, underlining a strong preference for warm water. Higher water intake was also recorded when heated water was provided to dairy cows during winter, in comparison to only ambient-temperature water being provided [17,19]. Additionally, Chen Zhaoui et al. (2015) reported a higher intake when heated water was administered to beef cattle [21].

Moreover, heating the drinking water can improve ruminal stability, enhancing the efficiency of feed processing by the ruminal microbiota and fermentation pathways. In fact, in the present study the ruminal environment was significantly more stable in the HW group: less time was spent per day under the reference thresholds for both ruminal pH and temperature. Several studies demonstrated that, in dairy cows, cold water intake significantly and drastically decreases ruminal temperature for extended periods of time: there is a deep and sudden decline that requires a period of time longer than three hours for recovery [16,17,18]. In a study by Bewley et al. (2008), the intake of 5.1 °C water decreased reticulorumen temperature by an estimated 9.2 °C. Up to 3.5 h was required for returning to the baseline temperature. Conversely, heated water (34.3 °C) significantly reduced the decrease in reticulorumen temperature and the time needed to return to baseline [16].

This drop in the ruminal temperature caused by the ingestion of cold water negatively altered the microbial activity, richness and diversity, thereby affecting the overall digestibility of the dry matter and all the fermentation patterns in in vitro studies [14,17]. The greatest effect was recorded in the cellulolytic population of the rumen. Rumen-based microbial attachment to fibrous substrates has been reported to be optimal at 39 °C; lower temperatures markedly reduce adhesion [15]. In fact, Petersen et al. (2016), in a study conducted in vitro and in vivo, reported that in vitro NDF degradability decreased from 41 to 14% when the water-bath temperature, which reproduced the rumen environment, decreased from 39 to 31 °C. However, these results were not confirmed in vivo because the duration of time in which the rumen temperature was below 38 °C was not sufficient to reduce NDF degradation [17].

For our study, the higher average pH values recorded and the shorter amounts of time spent at pHs under the selected thresholds of 5.8 and 5.5 can be explained by the better functionality of the cellulolytic bacteria resulting in a more stable ruminal environment. It is recognized that all fermentation patterns are positively influenced by a more stable ruminal environment, resulting in higher production of volatile fatty acids at the rumen level, which can explain the better growth performance found in the present study [36].

## 5. Conclusions

Administration of heated water at a constant temperature of 25° C instead of water at room temperature to beef cattle during their fattening period resulted in improved growth performance and in a more stable and healthier rumen environment, resulting in more efficient overall productivity. Sustainability and animal welfare are—and will increasingly become—the two fundamental pillars of the livestock sector. For this reason, more studies should be focused on developing new approaches which might enhance both issues, particularly for the meat production sector, where a change in consumer perception is needed. The possibility to optimize the physiological conditions of the rumen in livestock by administering heated drinking water during winter paves the way for increasing both sustainability and animal welfare.

## Figures and Tables

**Table 1:** Average water temperature values (T°C) detected in the RTW ^1^ group.

Month	Temperature, T °C
November	12.4
December	9.1
January	7.4
February	7.1
March	11.8
April	15.2
May	17.1

^1^ RTW-water at room temperature.

**Table 2:** Predicted diet composition and nutritional value, calculated by the rationing software (Plurimix).

	Arrival	Base	Fattening	Finishing
Days on Feed	**0–30**	**30–60**	**60–130**	**130–180**
Raw Material (kg)				
Corn silage	7.00	8.00	8.00	6.00
Corn meal	1.80	3.50	6.00	7.50
Soybean meal 44% CP ^1^	0.90	1.50	1.50	1.50
Wheat straw	1.20	0.90	0.90	0.90
Beet pulp	1.10	1.00	1.00	1.00
Minerals and Vitamins	0.20	0.20	0.20	0.20
Nutritional Values (% DM ^2^)				
DM ^2^	57.55	60.46	64.44	70.00
MJ ^3^	10.60	11.32	12.04	12.53
UFV ^4^	0.88	0.94	1.00	1.04
CP ^1^	12.16	13.53	12.55	12.37
Sugars	5.06	4.54	3.92	3.37
Starch	29.64	36.29	42.68	46.26
NDF ^5^	41.54	34.60	29.96	26.92
Fat	2.53	2.83	3.06	3.19
Ca tot	0.61	0.62	0.51	0.47
P tot	0.29	0.33	0.32	0.32

^1^ CP = crude protein; ^2^ DM = dry matter; ^3^ MJ = megajoule; ^4^ UFV = meat forafe units; ^5^ neutral detergent fiber.

**Table 3:** Growth performances and feed conversion rates of the two experimental groups.

Parameter	HW ^1^	RTW ^1^	SE ^2^	*p* Value
Live weight, kg (±ds)				
d_0_	405 (±38)	408 (±34)	6.045	0.59
d_90_	546 (±40)	544 (±34)	6.045	0.74
d_180_	719 (±59)	711 (±57)	6.045	0.28
ADG ^3^, kg/head/day				
ADG_0–180_	1.471	1.419	0.023	0.047
ADG_0–90_	1.560	1.513	0.033	0.160
ADG_90–180_	1.413	1.357	0.033	0.158
Feed intake, kg/head/day DM ^4^				
Average d_0_–d_180_	11.19	10.50	0.044	<0.001
**FCR**				
Average d_0_–d_180_	7.80	7.58	0.193	0.260
Slaughtering performance				
CCW ^5^, kg	421.29	413.56	4.45	0.084
CY ^5^, %	59.80	59.72	0.031	0.059

^1^ HW = constantly heated water at 25 °C; RTW = room-temperature water. ^2^ SE = standard error of the means. ^3^ ADG = average daily gain. ^4^ DM = dry matter; ^5^ CCW = cold carcass weight; CY = carcass yield.

**Table 4:** Ruminal parameters: ruminal pH.

Parameter	HW ^1^	RTW ^1^	SE	*p* Value
**Rumen pH**				
average	6.59	6.44	0.09	<0.001 ^a^
min	4.75	4.73		
max	7.86	7.64		
**Rumen pH, time under the thresholds, min/day**				
<5.8	20.32	31.04	0.56	<0.001
<5.5	8.69	12.39	0.36	<0.001

^1^ HW = constantly heated water at 25 °C; RTW = room-temperature water. ^a^ *p*-value for the month–group effect.

**Table 5:** Ruminal parameters: ruminal temperature (°C).

Parameter	HW ^1^	RTW ^2^	SE	*p* Value
**Rumen T°C**				
average	39.7	39.5	0.056	
min	31.9	32.5		<0.001 ^a^
max	41.8	42.6		
**Rumen T°C, time under the thresholds, min/day**				
<38 °C	35.50	66.76	0.15	<0.001
<37 °C	9.41	29.46	0.09	<0.001

^1^ HW = constantly heated water at 25 °C; ^2^ RTW = room-temperature water. ^a^ *p*-value for the month–group effect.

## Data Availability

The data presented in this study are available on request from the corresponding author.

## Supplementary Materials

The following are available online at https://www.mdpi.com/article/10.3390/ani11082218/s1, Table S1: Technical aspects of SmaXtec Premium Boluses
