# Supplementary material for: Effects of Heated Drinking Water on the Growth Performance and Rumen Functionality of Fattening Charolaise Beef Cattle in Winter
Source: Animals (Basel). 2021 Jul 27;11(8):2218. doi: 10.3390/ani11082218 (PMC8388547; doi:10.3390/ani11082218)
Supplement: Supplementary file 1 [file animals-11-02218-s001.zip › animals-1298216-supplementary.pdf]

Supplementary Table

Supplementary Table S1 Technical aspects of SmaXtec Premium Boluses

Technical Aspects	
**Dimension**	132 × 35 mm (length×diameter)
**Measurement interval**	10 min
**Measurement range (pH)**	pH 3 – 9
**Measurement range (temperature)**	0 °C – 80 °C
**Measurement accuracy (pH)**	Up to day 90 pH ± 0.2; up to day 150 pH ± 0.4
**Measurement accuracy (temperature at 39°C)**	± 0.05°C
**Measurement resolution (pH)**	pH 0.01
**Measurement resolution (temperature)**	0.01 °C
**Activity index**	0 to 100
**Measurement duration pH measurement**	150 days (5 month)
**Battery life (temperature and activity measurement)**	Up to 4 years
